# Miscellaneous

**Published:** 1875-03

**Authors:** 


### Miscellaneous.

### ON TAKING PLASTER IMPRESSIONS.

BY ANDREW WILSON, ESQ.

Judging from the general tone of the remarks made on the subject of plaster impressions, at the December meeting of the Odontological Society, as reported in the last number of this Journal, one of the chief (if not the chief) objections was the unpleasantness of the plaster flowing into the mouth, and especially over the throat.

Having suffered very frequently from this happening, and thoroughly appreciating the advantage of getting a trustworthy impression, I was very glad when, three years ago, assistant proposed the use of soft modeling clay as a barrier to stay the egress of the plaster at the palate. I have ever since used it, and find that it almost, if not entirely, gets rid of that drawback in taking plaster impressions.

For the benefit of those who may wish to give it a fair trial I will describe my mode of proceeding, taking an upper suction case as an example. First take the impression in Godiva, cool it thoroughly, pare away all superfluous bulk, and with a sculptor cut away freely the composition round all remaining teeth, and deepen as well as widen the parts, giving the alveolar ridge, cutting most freely where any part of the ridge is soft or pendulous. The result of this is that the only portion of the original surface left is the palate. I then cut a groove with a flat-pointed graver, along the back part of the palate, close to the edge (to prevent the displacement of the clay under pressure), and if the surface seems to require it roughen the surface of the palate by scoring it with a knife. I now take a little *soft* modeling clay and build a wall on the groove, varying in height from one quarter to one eighth inch. The tray is now ready for filling with plaster, and when introduced into the mouth the back part is first brought to its position in contact with the palate, which presses the clay into its own form, and thus preventing the plaster from escaping in that direction drives it forward. All superfluous plaster escapes under the lip (or against the cheek), causing almost no uneasiness, while being useful as assisting you to judge as to when to remove the impression, as plaster sets more quickly in the mouth than in the basin. The forward movement of the plaster in the tray has another and very important advantage, as you obtain a much sharper impression of the *front* part of the palate and rugæ than you would otherwise obtain.

For impressions of the lower jaw I form a wall along the whole inner edge, much stronger, as here it acts not only as preventing the escape of the plaster, but also as damming out the saliva.

In cases where any of the remaining teeth are *very* much necked or decayed, or lie at a *great* angle to the others, and the impression is not likely to break at them, I make up the teeth to draw more easily with soft clay.

As regards the plaster, I prefer using it cold, for two reasons: it is less likely to cause nausea, and there is not that tendency to granulate which I have always found takes place when warm water was used.

Of chemicals to produce quick-setting, I find Fletcher's alum in solution, in the proportion of one ounce to the imperial pint, and colored with a little burnt umber, answers best; from two to three minutes sees it hard enough for removal.

Common salt has the great disadvantage of being deliquescent, and soak the impression as you may, the model from it is inclined to get damp, telling against it when in the molding sand.

The objection that saline ingredients produce cold, of course only applies when the salt is added to the wet plaster as a *solid* (powder), when it acts as in freezing mixtures.

Saline solutions act in that respect just as pure water of the same temperature would do, and as you can always use a solution of uniform strength in making up your plaster, you can look for equally uniform results.

I think it an advantage to make the solution with water which has been boiling for a short time, as the water being thus freed from all air in solution, there seems less liability to air-blows in the plaster.

For small impressions, say not going beyond the first molars, or involving much of the palate, I only line the tray irregularly with the Godiva (so as to prevent the plaster leaving the tray, unless heat be applied), clay rarely being required, as with Fletcher's solution of plaster is slightly viscid and much less inclined to run than with either salt water or pure water.

Of course it requires some practice to give confidence and make it (in most cases) easy, but the greater the difficulty in taking a plaster impression in any case, the greater the advantage, I may say the necessity, of having one, all other materials yet employed, be it Godiva, Stent, or wax, giving miserable apologies for impressions in such cases. In conclusion, the best results with plaster impressions can only be obtained or looked for where the principal himself is willing to be at the trouble, not only cementing *in situ* all pieces of importance which may break off in removing it from the mouth, but also of separating the model from the impression afterwards.—*British Journal of Dental Science.*

---

### INJURY OF THE FACE.

BY J. S. THURMAN, M. D., OF CEDARVILLE, MO.

I am prompted to report the following case, on account of its rarity in country practice. In Southwest Missouri physicians seldom meet with cases requiring immediate surgical appliances.

I was called in great haste, on the 5th of January last, to see Elois Acock, aged twenty-four years. On arrival I found him lying on the floor in a semi-conscious state, with copious hemorrhage flowing from the mouth and nose. I learned, upon inquiry, that he was passing near the heels of a horse, and slapped it with his hand, at which the animal became frightened, and kicked with all its force, striking patient on right side of the superior maxillary bone and mouth. I found, upon examination, the upper jaw to be fractured, in a line commencing just behind the last molar tooth, and passing across the malar process to the lateral boundry of the anterior nares, and then down between the canine and incisor teeth. On manipulation of the left side of the upper jaw, I detected mobility and crepitation, but was unable to determine the line of fracture; apparently all the bones of the face were detached and the soft parts terribly contused.

The swelling soon closed his eyes; then he was a horrible looking object. There was a gash cut in the lower lip, I presume by the shoe of the horse, three-fourths of an inch in length. Reaction came on in three or four hours. The hemorrhage was soon controlled by the application of cold water. Several small spicula of detached bone were removed with dressing forceps. The fractured part was then adjusted by introducing my finger into his mouth and placing and holding the detached portion until a cork was placed between the first molars on each side, and the lower jaw brought firmly up, which held the cork in its place; a bandage was then applied, such as used in fracture of the inferior maxillary. The gash in the lip had previously been united with sutures; the other bones were replaced as far as possible. Patient was then placed in bed, and the mouth and nose ordered washed with solution of carbolic acid four times per day, and fluid nourishment. The first twenty-four hours patient was very restless, after which he became more quiet and rested well. I removed the bandage on the 14th day, and found union perfect. Some discharge from nose. Four weeks after, I saw patient at my office; there was no discharge from the nose and he said he was satisfied with his jaw, but should have been glad of a few more teeth.—*Reporter.*

### TORSION AND REPLANTATION.

Within the last few months numerous communications have appeared in the dental journals in reference to these operations. It is noteworthy how generally they are treated of as simple mechanical procedures. But assuredly elements beyond the mere mechanical enter into and should determine the wisdom of all such operations. Whoever deals with vital tissues, will learn that "circumstances alter cases." An operation which may in one instance be perfectly justifiable, and suggestive only of good results, may, in another case, be contraindicated so strongly that, if evil consequences ensue, the verdict would be compelled that ignorance or carelessness was manifested by the practitioner.

In cases of injury or of abnormal growths threatening life or limb without surgical interference, the hazard of untoward results must be accepted by the operator; the choice of evils as well as uncertainties must be made; but where the problem is merely the correction of an irregularity, involving only the question of improved appearance, the risk of consequences more to be dreaded than the existing condition should be taken in to the account.

It is a curious and instructive lesson, that Nature's toleration of heroic treatment seems to be in proportion to the absolute necessities of the case. Thus tetanus, and perhaps erysipelas, are more apt to follow upon slight than upon severe operations. The deaths from anæsthetics are startlingly associated with minor operations. A tight boot, the clumsy paring of a corn, a puncture by a tack, a scratch of a fellow-sleepers's nail upon the skin, and other like trivial matters, have resulted not unfrequently in serious disturbances, and even in death.

An injury of a member sought to be retained is generally more troublesome than the results of amputation, and torsion, it is not hard to believe, it is vastly more liable to produce mischief than extraction would be.

There are wonderful differences of susceptibility in different individuals, and equal differences in their reparative power. There is also in the same individual, at different times, a greater or less power of resistance to deleterious influences. Not only therefore should the idiosyncracies of the individual be considered, but the existing systemic condition. Every one is liable at times to a general lowering of the resistive capacity, in which state, as patients often express it, "every scratch becomes a sore." The probabilities of success in torsion or replantation, under such circumstances, would evidently be much less than in normal condition, when union by first intention takes place even after severe wounds.

The ability to estimate constitutional predisposition or systemic degeneration would be of essential service in determining the likelihood of success or failure in such operations as those alluded to. It must be granted that it is not always possible to be so skillful in diagnosis as surely to foretell success, but on general principles the chances may be approximately estimated.—*Dental Cosmos.*

